# Supplementary material for: Predicting the diversity of photosynthetic light-harvesting using thermodynamics and machine learning
Source: PLoS Comput Biol. 2025 Mar 11;21(3):e1012845. doi: 10.1371/journal.pcbi.1012845 (PMC11896073; doi:10.1371/journal.pcbi.1012845)
Supplement: Attachment — Submitted filename: C_Gray_evo_antenna_resubmission_response.docx [file pcbi.1012845.s002.docx]

**Comments to the Authors:
Please note here if the review is uploaded as an attachment.**

Reviewer #1: The paper is very interesting and leverages years of research on natural light-harvesting in different ecological niches to open an unexplored, exciting research line.
I would recommend, for clarity, to add to the manuscript the following information:
-in the Intro, please explain for cooler and smaller M-dwarf stars what is the emission range, for clarity.

- **This is an important point to note, thank you. We have added text to explain that M-dwarfs emit more at longer wavelengths and can have emission maxima at ~1um. We also show the spectrum in Fig. 6 for reference.**

-line 88-89, please explain why being oxygenic antenna you choose specifically 5 ps and provide references

- **We have added a paragraph briefly explaining the various processes occurring in the oxygenic reaction centre and the timescales involved, along with our reasoning for choosing 5ps for the “trapping” time and relevant references. In essence, we define *trapping* as the process of primary charge separation of the exciton once it has arrived on the RC. It does not include migration of the exciton to the RC nor the subsequent charge-stabilization, acceptor reduction and donor oxidation steps. What matters in our model is the (rather fast) timescale for when an exciton ceases to be an exciton and then energy transfer gives way to electron transfer.**

-lines 138-146: explain how your choice of identical branches differ from natural supercomplexes and how, choosing them identical, it might limit the model.

- **We have explained that our choice of identical branches is motivated partly by computational considerations, and partly by the fact that many real antenna systems are modular or symmetric in this way, but noted that asymmetric antenna systems do exist; their behaviour would not be captured by this model and would require further work.**

-Figure 3: the bottom plots are not clear, what are the different colors of the histograms referring to? also, I cannot find a comment in the text about this part of the figure. Also, and how does the distance from the RC from your models compare with the actual ones of natural supercomplexes? I cannot find it commented even in the text but I might have overlooked.

- **We have removed all the histograms from the main text and replaced them with tables detailing approximate pigment composition. The histograms are now in the Supplementary Material and now have legends on them to make them more readable at a glance. We have also added a comment explaining that the physical locations of the pigments are not considered microscopically; we only assume that they are close enough to each other (and possibly the RC) to exchange energy. The model is best thought of in terms of thermodynamics and topology rather than a real space model. We assume a separation of timescales in which pigment states in the same sub-unit exchange energy very rapidly (instantaneously) and neighbouring subunits exchange energy more slowly (~10 ps). We explain in the text that ‘subunits’ do not necessarily represent distinct pigment-protein complexes, merely strongly-coupled-clusters of pigment.**

-the method references another method used by the author in a paper submitted for review, in the references. what is the difference between the two papers?

- **This method presents an entirely new genetic algorithm for simulating evolution of the antennae along with an extension of the previously published theory, which allows for (in principle) arbitrarily larger antennae with arbitrarily complicated absorption lineshapes. In effect the previous (now published) paper validates the underlying thermodynamic model but has no evolutionary component. This work is based on the evolutionary model.**

Reviewer #2: The manuscript by Gray et al. is an interesting exploration on the in silico evolvability of photosynthetic antenna structures for different solar spectra. The authors generated a coarse, but detailed enough, model for light harvesting and applied an evolutionary model to allow the systems to respond to the imposed light environments. Overall, the article is well written and of interest for the discussions arising from their results.

There are a few points of criticism/clarification that I belive would improve the manuscipt.

In general, all of the simulations were performed under 'high light'. Could the authors clarify what they mean by this in relative photon flux or other terms? Have simulations been performed at medium (say ~50% high light) and/or low (~5% of high light)? That would be interesting to compare the pigment composition, as well as pigment and subunit number of the antenna.

- **This is a very good point, and we would like to thank the reviewer for this comment. In the process of addressing this we discovered a mistake in our treatment of the overlap integrals which modify transfer rates between blocks, leading to a global underestimation of transfer rates; this has now been fixed and we have performed our calculations again, finding no significant differences in pigment composition but an increase in overall antenna efficiency. We have also performed simulations for “medium” (300 mu mol m-2 s-1, corresponding to ~30-50% of the high light simulations depending on light input) and “low” (50 mu mol m-2 s-1) light simulations, with results presented in Fig. 3 for a range of cost values, and included an estimate of the amount of PAR in full sunlight to make clear that the AM1.5 spectrum is extremely bright (and pointed out that we neglect photoprotective mechanisms such as NPQ).**

  -Line 11: I do not belive that the parent start is the most obvious source of energy for life. One only needs to look at Earth, where LUCA would not have been capable of photosynthesis (see https://doi.org/10.1038/s41559-024-02461-1 for a recent estimate of ~4.2 Ga for LUCA versus https://doi.org/10.1111/gbi.12322 generating an estimate of ~3.4 Ga for the origin of the Type II reaction center proteins). This does not change the relevance of the manuscript, but does greatly alter the importance of photosynthesis for establishing life on a planet.
- **It is true of course that the parent star is not the only source of energy for life. We have edited the section of the paper in question, adding a discussion of our reasoning for investigating photosynthesis specifically as a general source of energy on any planet which may lead to detectable biosignatures.**

  Lines 17-18: The Em for the half reaction of water oxidation is +0.82 meV at pH 7.0, while P680+ has a potential of ~1.2 eV which provides an over-potential for water oxidation. Shifting the wavelength of P to 727 nm as in the chlorophyll f PSII still results in an overpotential for water oxidation. The overpotential may be necessary for the biological dissipation processes within the reaction center itself (not considered in this work), however, red shifting the reaction center potential to still achieve water oxidation is fully feasible. It may be worthwhile for the authors to include these potentials to better highlight their point.
- **We have mentioned that under standard conditions there is an excess of energy available for water oxidation, but since the focus of this paper is on the evolution of the antenna, and we want to be as general as possible, we have refrained from including specific reaction centre potentials. As mentioned in the text, a much more detailed model of the combined antenna-RC supersystem is the focus of our next work, and there we will treat RC redox potentials more quantitatively. We have also added a separate results subsection with additional simulations performed under far-red light with a far-red adapted RC at 727nm, of which more below. We note that addressing this comment has, in our view, substantially improved the paper.**

  Lines 49-50: For most antenna, describing them as energy funnels is not particularly true. This is likely only the case for phcobilisomes which clearly act as funnels from higher to lower energy pigments. In most antenna, however, the pigments are near isoenergetic with dozens (or hundreds) of pigments within a few nanometers of identical absorption.
- **We agree that describing all antennae as energy funnels is an unhelpful simplification and have addressed this in the text. In addition, we allow the formation of completely flat antennae (e.g. completely composed of Chl a) within the algorithm used in this work, and have noted this in the text.**

  Line 55-57: "Lastly, the antenna should not be so large and complex that its creation and maintenance leaves little surplus energy for growth, reproduction, etc." I would suggest the authors clarify if large and complex are separate entities or one. For example chlorosomes are large (hundreds of thousands of chlorophyll molecules) but one could argue not complex.
- **This is true; we meant large, rather than complex. We have fixed the wording. In essence our cost parameter chi is very crude, simply representing a number of electrons-per-antenna-pigment that must be reinvested into maintaining the antenna. We discuss the meaning of such a ‘cost’ and suggest avenues of further work.**

  Lines 83-84: Why were RC dissipative processes neglected? If modeled are the results the qualitatively the same? Or does it become too cumbersome to model? Please clarify.
- **We’ve clarified that in this work we do not consider dissipative processes within the RC since we primarily focus here on the effect of the antenna, and that we simply assume dissipative processes in the reaction centre are out-competed since otherwise the antenna would not function properly. A more quantitative description of RC processes is the focus of further work.**

  Bottom of page 4 (should be lines 85-87 but are not numbered): "Open" and "Closed" are commonly used terms to describe the reduction state of reaction centers. it seems unnecessary to use alternative nomenclature.
- **We have changed the wording here, thank you.**

  Figure 1: Figure 1 is not referenced in the text at all except for 1B. The ordering of the panels is also slightly confusing and could be shuffled to make the current letters A-E as: A, B (top), C (middle), D, E (bottom) and maintain alphabetic ordering left to right rather than top to bottom then top to bottom again.
- **Thank you - this ordering is clearer and we have changed the figure to reflect it. The lack of references to the other subfigures was an omission and they are now all referenced.**

  Figure 1B: These are the absorption spectra of pigments in solvent, presumably? Please clarify.
- **These are indeed for isolated pigments in solvent and we have stated this in the text. The chlorophyll/bacteriochlorophyll spectra are all specifically in pyridine, the cyanobacterial pigments are simply “in solution” and digitised from elsewhere. We have also added the relevant references.**

  Line 86-87: The rate of trapping varies for open vs closed PSII. Please explain where the 5 ps trapping time came from, as the cited paper (Broess et al.) appears to only reference 5 ps as a kinetic needed to account for the excitation light. Most reported PSII trapping rates are 2 orders of magnitude larger.
- **This is a level of complexity that we think is beyond the scope of the current work; in this work we aim to be as general as possible, and hence we simply assume that the RC can trap excitations. Our reasoning is simply that the timescale for the exciton converting to a metastable charge-separated state (i.e., for oxygenic photosynthesis, formation of the initial radical pair) must happen on (approximately) the same timescale as energy transfer between antenna subunits, otherwise the exciton could easily hop back out of the reaction centre, and hence choose a “trapping” timescale comparable to this. We note that ‘trapping’ simply means the timescale of the exciton, having already arrived at the RC, undergoing the first charge separation step. In effect it ceases to be an exciton and energy transfer gives way to electron transfer. All subsequent charge separation steps (and dissipative steps) are renormalized into an abstract ‘output rate’. We have added a discussion of this point in the Methods section along with additional references highlighting this complexity.**

  Line 197: please correct (ref)
- **This was a note left in the text and we have replaced it with the intended citation.**

  For all figures containing solar spectra and results, please scale the incident solar spectra to be visible in the panel. This allows some understanding and comparision between different situations. See Fig. 2C, Fig. 5B

  For Fig. 2C, is the intensity (y-axis) refering to the absorption spectra or the indicent light? This could be made clearer on all figures with multiple parameters plotted.
- **These are both very good points. We have replotted all the figures to include proper y-axis labels; the y-axis denotes the intensity of the incident light, while for the absorption spectra we have simply normalised their peaks to 1. In order to make the figures as legible as possible we have kept consistent y-axis limits throughout and increased them to make the incident spectra more visible.**

  Many of the supplemental figures are not referenced in the text of the manuscript and should be.
- **We have changed or added many new supplementary figures and tables; these are all now referenced in the main text. Thank you.**

  Figure 3: The dotted spectra represent different complexes from PSII, phycobilisomes, and whole cells. However, many of the individual (PSII) and combined (PSII and phycobilisome) spectra are available, especially rather than using whole cell spectra. Clarifying these spectra could help, as well as clarifying that the authors are showing the similarity (or lack thereof) to antenna rather than the antenna-PSII complex. Please also scale the dashed line in D to fit on the graph.
- **We have in general changed the experimental spectra shown alongside the simulated absorption curves to represent antenna-RC absorption where possible. The red light is now compared to BBY particles from spinach rather than just PSII; the PCC 6803 and *L. glaciale* spectra used for comparison in the main text are for whole cells.**

  Line 278: Type III is gererally used for complementary chromatic acclimation, not group III.
- **This has been fixed, thank you.**

  Figure 5: It would be useful to also display the antenna composition for the anoxygenic antennas
- **In general we have removed the bar charts from the main text and replaced them with tables showing approximate pigment composition; the bar charts are now in the supplementary material. This is done mainly to keep the number and size of figures down.**

  Line 332: '...utilising a Chl a antenna' I think would be better phrased that on other planes an organism could exists using antenna with similar spectral properties as chlorophyll a.
- **We have reworded the sentence to reflect this, thank you.**

  The far-red light evolution is interesting for its lack of evolving. However, in these organisms the phycobilisomes are also still present though wavelength shifted, with altered PSII trapping rates as well (see Mascoli et al. Nature Communications 2022). If the trapping rates are altered in this environment, can an antenna evolve? For non-experts, it would help if the authors described how their results show that the system did not evolve.
- **As mentioned, we do not explicitly consider changing of internal trapping rates within the reaction centre, since this will be the focus of further work. We have added a note in the text explaining that we define non-evolution by our algorithm failing to find any solutions with positive fitness values.**

  It is interesting that chlorophyll d is so rarely represented in the antenna. It seems to only be prevalent in far-red light when the cost is low. However exclusively chlorophyll d antenna are found in Acaryochloris species. If the reation center is shifted to 727 nm does chlorophyll d become used in any radiation spectrum?
- **As mentioned above, we have performed an additional set of simulations for differing amounts of far-red light with a redshifted RC as in FaRLiP, with results shown in Fig. 4. These antennae do evolve and in fact we find a shift from Chl a and b to Chl d and finally Chl f as the availability of visible light decreases. This part was also motivated by this reviewers comment and we note, again, that this has substantially improved the paper.**

Reviewer #3: The paper by Gray et al. presents a fascinating and exciting study of the possible biodiversity of photosynthesis on exoplanets hosting different environments than Earth. The authors construct a physical model of photosynthesis based on the known physics of photosynthetic antennae on Earth and simulate the evolution of photosynthetic antennae under different illumination conditions and different costs of antenna production using a genetic algorithm. Interestingly, they reproduce roughly and qualitatively the properties of photosynthetic antennae in various environments on Earth, which gives them the confidence to attempt predictions for extra-terrestrial environments.

The physical model the authors use for photosynthesis is a solid one, and it probably works because the most important steps of energy transfer in photosynthesis are based on weak coupling between pigments or whole antennae. It is, therefore, possible to estimate the rate by the formula (4) of the paper. The authors present a general model of exciton transfer and restrict it to a single excitation regime valid for photosynthesis on Earth. They find a range of production costs that gives them reasonable results - i.e. non-trivial antennae and with this range they study the conditions characteristic of exoplanet environments.

The study is a very valuable one because it provides physical backing to hypotheses about photosynthesis evolution on exoplanets. It is easy to see that this study is open to many ways of generalization and modification as it provides just a first step in what may become a fields of its own. In fact, an evolutionary study like this can provide some sanity check to speculations about the optimization of photosynthesis by tuning various details of the antenna which are often suggested by theoretical studies. Here the model is simple, robust, and void of weird quantum effects, yet it captures essential characteristics of real photosynthesis. I wholeheartedly recommend this paper for publication.

I have only one question, which the authors may use to optionally revise their manuscript. I was missing the information about the production cost for the existing organisms. It would boost the confidence of the reader if this number agreed with the simulation results.

- **Unfortunately, it is basically impossible to come up with a single quantitative number representing production cost for real organisms; we have here condensed down many different considerations, such as the cost of synthesising pigments, the cost of maintenance, the cost of supporting non-photosynthetic structures, and physical space constraints all into one number. This is done because it is impossible to know *a priori* exactly what the broader organisms would look like, because simulating entire real organisms is far too difficult, and because for computational reasons we need to constrain the size of the antennae to some extent. We have added a paragraph explaining this in the discussion of the genetic algorithm.**

Description of changes:

- We have added new simulations for medium and low light illumination and modified the existing results subsection of different terrestrial light environments to include these results.
- We have added a new results subsection in which we present results of simulations on low intensities of visible light with a far-red adapted reaction centre
- We have modified the results subsection on contingent evolution to include multiple cost values
- We have replaced pigment composition histograms with tables
- We have replaced the supplementary material figures with extra tables and new figures along with histograms showing detailed antenna composition for all of the results presented in the main text
- We have made various small textual revisions in accordance the reviewers’ comments.
